# Synthesis of High‐Quality Graphene and Hexagonal Boron Nitride Monolayer In‐Plane Heterostructure on Cu–Ni Alloy

**DOI:** 10.1002/advs.201700076

**Published:** 2017-05-19

**Authors:** Guangyuan Lu, Tianru Wu, Peng Yang, Yingchao Yang, Zehua Jin, Weibing Chen, Shuai Jia, Haomin Wang, Guanhua Zhang, Julong Sun, Pulickel M. Ajayan, Jun Lou, Xiaoming Xie, Mianheng Jiang

**Affiliations:** ^1^ State Key Laboratory of Functional Materials for Informatics Shanghai Institute of Microsystem and Information Technology Chinese Academy of Sciences Shanghai 200050 P. R. China; ^2^ CAS Center for Excellence in Superconducting Electronics (CENSE) Shanghai 200050 P. R. China; ^3^ Department of Materials Science and NanoEngineering Rice University Houston TX 77005 USA; ^4^ School of Electronic, Electrical and Communication Engineering University of Chinese Academy of Sciences Beijing 100049 P. R. China; ^5^ School of Physics and Electronics Central South University Changsha 410083 P. R. China; ^6^ State Key Laboratory of Molecular Reaction Dynamics Dalian Institute of Chemical Physics Chinese Academy of Sciences Dalian 116023 P. R. China; ^7^ School of Physical Science and Technology ShanghaiTech University Shanghai 200031 P. R. China

**Keywords:** chemical vapor deposition, Cu–Ni alloy, graphene and *h*‐BN in‐plane heterostructures, high quality

## Abstract

Graphene/hexagonal boron nitride (*h*‐BN) monolayer in‐plane heterostructure offers a novel material platform for both fundamental research and device applications. To obtain such a heterostructure in high quality via controllable synthetic approaches is still challenging. In this work, in‐plane epitaxy of graphene/*h*‐BN heterostructure is demonstrated on Cu–Ni substrates. The introduction of nickel to copper substrate not only enhances the capability of decomposing polyaminoborane residues but also promotes graphene growth via isothermal segregation. On the alloy surface partially covered by *h*‐BN, graphene is found to nucleate at the corners of the as‐formed *h*‐BN grains, and the high growth rate for graphene minimizes the damage of graphene‐growth process on *h*‐BN lattice. As a result, high‐quality graphene/*h*‐BN in‐plane heterostructure with epitaxial relationship can be formed, which is supported by extensive characterizations. Photodetector device applications are demonstrated based on the in‐plane heterostructure. The success will have important impact on future research and applications based on this unique material platform.

Monolayer graphene and hexagonal boron nitride (*h*‐BN) are two representative 2D crystals possessing similar atomic arrangements and lattice constants, but with completely different electronic properties.^1^, ^2^ Graphene/*h*‐BN monolayer in‐plane heterostructure, formed by merging the two materials into a single atomic layer, has attracted considerable attentions owing to its promising electronic applications. It has been both theoretically predicted and experimentally verified that the band gap can be opened and tuned by embedding *h*‐BN domains in graphene layer.^3^, ^4^, ^5^, ^6^, ^7^, ^8^ Some electronic applications were demonstrated based on patterned graphene/*h*‐BN in‐plane heterostructures.^9^, ^10^ Exciting physical properties were also predicted for the in‐plane heterostructure, further revealing its potential values in scientific research and future applications.^11^, ^12^, ^13^

Sequential growth of graphene/*h*‐BN on catalytic metal surfaces via chemical vapor deposition (CVD) has been extensively studied to form in‐plane heterostructure.^9^, ^10^, ^14^, ^15^, ^16^, ^17^, ^18^, ^19^, ^20^ Templated growth of *h*‐BN starting from CVD graphene edges on copper has been realized with lattice coherency of the two crystals.^14^, ^16^ However, defects may form in graphene domains due to its relatively low chemical stability during the *h*‐BN growth. Methods in the reversed order were also reported by growing *h*‐BN first, followed by graphene growth, either on patterned *h*‐BN or isolated *h*‐BN grains. However, imperfections still could be observed in graphene.^9^, ^15^, ^21^ Syntheses of graphene/*h*‐BN in‐plane heterostructure have also been achieved by etching regrowth or chemical conversion methods after growing a continuous *h*‐BN film.^18^, ^20^ The etching regrowth or chemical conversion was found to start preferentially at the defective sites of *h*‐BN, while the long growth process also creates many defects in *h*‐BN lattices.

Here we report the successful synthesis of large‐area graphene/*h*‐BN in‐plane heterostructure by sequentially growing *h*‐BN single‐crystals and graphene grains on Cu–Ni alloy. The alloy's enhanced ability in decomposing polyaminoborane residues improves the cleanliness of the alloy surface during *h*‐BN growth.^22^ It is found that graphene nucleates at the corners of the triangular *h*‐BN grains and follows the lattice orientation of *h*‐BN, indicating the epitaxial relationship between the two crystals. As the growth of graphene on Cu–Ni alloy occurs in isothermal segregation,^23^ it not only speeds up the growth of graphene but also greatly reduces the damage to *h*‐BN lattices. As a result, extensive characterizations confirm that both graphene and *h*‐BN are in high qualities. Photodetector devices were demonstrated by integrating monolayer 2D semiconductor materials to the in‐plane graphene/*h*‐BN heterostructure. The method will facilitate both future 2D electronics and physical studies based on large‐area high‐quality graphene/*h*‐BN in‐plane heterostructure.

In this work, a two‐step low pressure chemical vapor deposition (LPCVD) method was employed. Cu–Ni alloy foils containing 15 atom% Ni (Cu_85_Ni_15_) were used as the catalytic substrates, and ammonia borane (H_3_BNH_3_) and methane (CH_4_) were chosen as the precursors for *h*‐BN and graphene growth, respectively. The time dependence of experimental parameters is given in Figure S1 (Supporting Information), while the experimental details for *h*‐BN growth were described in our earlier report.^22^

The detail of processes from *h*‐BN single‐crystals to graphene/*h*‐BN in‐plane heterostructure on Cu–Ni surface is schematically illustrated in **Figure** **1**a. And scanning electron microscopy (SEM) images showing the corresponding stages are given in Figure 1b–e, respectively. As shown in Figure 1b, triangular *h*‐BN single‐crystals with the lateral size of 40–50 µm were successfully synthesized on Cu–Ni surface after 40 min of growth. These *h*‐BN single‐crystals were mainly monolayered as demonstrated by our earlier report.^22^ By introducing methane into the reaction chamber for 30 s after cutting off the *h*‐BN precursor, dendritic graphene grains began to form. As shown in Figure 1c, the graphene appears darker, and it seems that graphene growth starts from the corners of the triangular *h*‐BN grains. With 60 s of growth, the graphene grains grew larger and merged with each other, as shown in Figure 1d. Finally, by further extending the methane‐supply time to 75 s, continuous graphene/*h*‐BN in‐plane heterostructure formed with triangular *h*‐BN grains embedded in the graphene film, as presented in Figure 1e and Figure S2 (Supporting Information).

**Figure 1:**
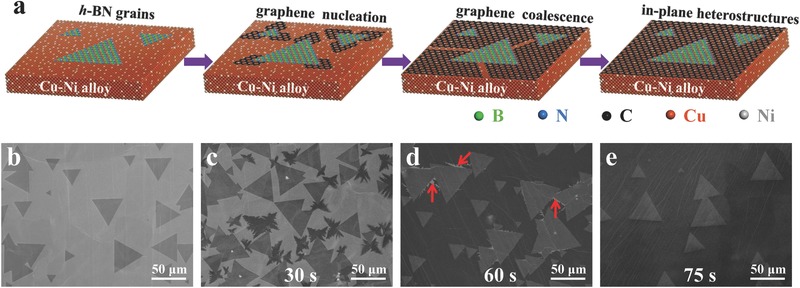
Formation of graphene/*h*‐BN in‐plane heterostructure on Cu–Ni alloy. a) Schematics showing the process from *h*‐BN grains to graphene/*h*‐BN in‐plane heterostructure. b) Typical SEM image of *h*‐BN grains after 40 min growth. SEM images of the *h*‐BN grains after subsequent graphene growth for c) 30 s, d) 60 s, and e) 75 s, respectively. The bright areas indicated by red arrows in (d) are naked Cu–Ni surface.

Experiments were carried out to investigate the influence of nickel introduction on the formation of the high‐quality in‐plane heterostructure. As shown in Figure 1c, graphene nucleates preferentially at the corners of the *h*‐BN triangles on Cu–Ni surface. Such preferential nucleation can be more convincingly justified by investigating graphene growth on Cu–Ni substrate with smaller *h*‐BN grains as presented in **Figure** **2**a (the growth time of *h*‐BN was shortened to 20 min as compared to 40 min in Figure 1b, making the *h*‐BN grains smaller but with much lower density). Dendrite‐shaped graphene nucleus was observed at each corner of *h*‐BN grain but nowhere else on the substrate. With extension of growth time, hundred micrometer sized graphene grains grew, coalesced and eventually resulted in a continuous film with embedded single‐crystal *h*‐BN grains, as shown in Figure 2b,c, respectively.

**Figure 2:**
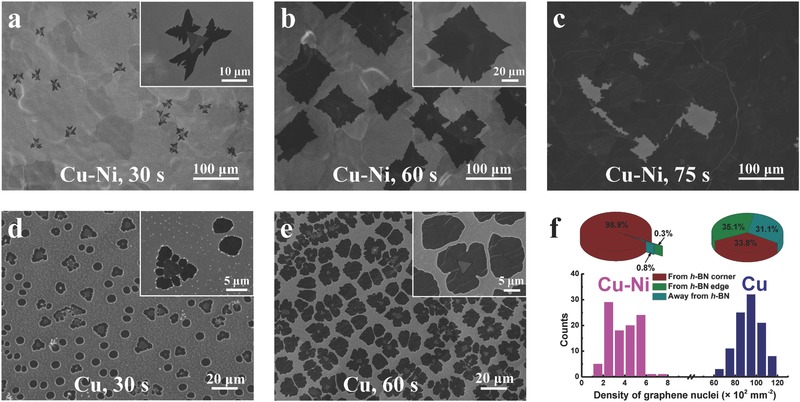
Substrate‐mediated graphene nucleation and growth through *h*‐BN grains. a–c) SEM images of Cu–Ni surface after the sequential growth of *h*‐BN for 20 min and graphene for a) 30, b) 60, and 75 s, respectively. d,e) SEM images of copper surface after the sequential growth of *h*‐BN for 5 min and graphene for d) 30 and e) 60 s, respectively. The inset in (a), (b), (d), and (e) shows the corresponding enlarged image of the graphene/*h*‐BN grains under each circumstance, respectively. f) Histogram of graphene nucleation density on Cu–Ni (magenta) and Cu (navy) surfaces with *h*‐BN grains, with the pie charts given above showing the distribution of the three types of graphene nuclei on each substrate, respectively. The statistical data are obtained from total 100 areas of 200 µm × 200 µm on each substrate.

For comparison, the experiments were repeated on copper foils with similar processing condition, and the results are presented in Figure 2d,e. Besides showing a much higher nucleation density (see Figure 2f), only one‐third of the graphene grains are observed to nucleate at the corners of *h*‐BN. Another one‐third of graphene grains grow along the edges of *h*‐BN. And the remaining one‐third of graphene grains appear on the copper surface away from *h*‐BN grains. Such randomness of graphene nucleation on copper is in qualitative agreement with literature studies.^15^, ^21^

Raman spectroscopy was utilized to characterize graphene/*h*‐BN in‐plane heterostructures grown on Cu and Cu–Ni substrates. **Figure** **3**a,b shows the optical images of the graphene/*h*‐BN in‐plane heterostructure grown on Cu–Ni and copper surfaces and transferred onto Si substrates with a 90 nm SiO_2_ capping layer, respectively. Figure 3c gives the Raman spectra taken from dotted areas in Figure 3a,b. It is worth noting that, due to its extremely weak signal intensity, *h*‐BN (cyan dot) requires an integral time 20 times longer than that for graphene (magenta, olive, and violet dots). The peak observed at 1370 cm^−1^ corresponding to E_2g_ phonon mode indicates that the *h*‐BN grain (cyan dot) is well crystallized after graphene growth.^24^ For the graphene grown on Cu–Ni (magenta dot), the intensity ratio of 2D band (≈2686 cm^−1^) to G band (≈1585 cm^−1^) is 2–3, and the full width at half maximum of the 2D band is about 32 cm^−1^, confirming that the graphene grains are monolayered and with high quality.^25^, ^26^ The D band (≈1348 cm^−1^) intensity of graphene grown on Cu–Ni is more than one order of magnitude lower than that of graphene grown on copper foil (Figure 3d,g). The contrasting results shown in Figure 3d–i give strong evidences that the quality of graphene/*h*‐BN in‐plane heterostructure grown on Cu–Ni is significantly higher than that grown on copper foil.

**Figure 3:**
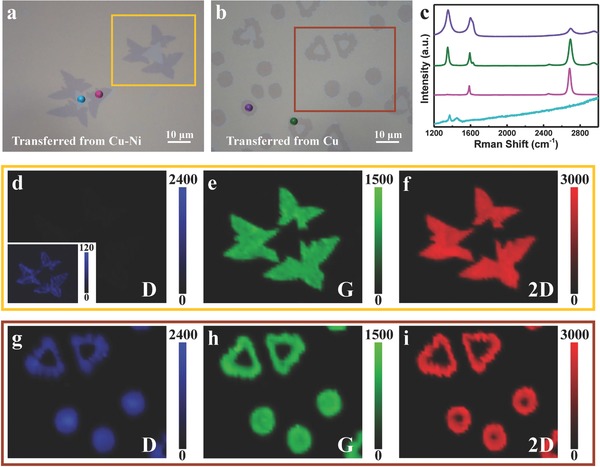
Raman characterization showing the quality difference between graphene/*h*‐BN grains grown on Cu–Ni and those grown on Cu. a,b) Optical images of the graphene/*h*‐BN grains grown on a) Cu–Ni and b) Cu after transferred onto 90 nm SiO_2_/Si substrates. c) Raman spectra taken from the marked areas with corresponding color dots in (a) and (b). The corresponding Raman maps of d) D, e) G, and f) 2D bands obtained from the region marked with yellow rectangle in (a). The inset in (d) shows the corresponding high‐contrast D map with a much lower intensity scale. Raman maps of g) D, h) G, and i) 2D bands obtained from the region marked with brown rectangle in (b).

We would attribute the much decreased nucleation density as well as the highly improved quality of graphene to the higher catalytic power of Cu–Ni in decomposing polyaminoborane, a polymeric residue precipitated during *h*‐BN growth.^22^, ^27^, ^28^, ^29^ As shown in Figure 2d, the polymeric residues seem to facilitate the graphene nucleation on the copper foil, resulting in the randomness of graphene nucleation after *h*‐BN growth. The polyaminoborane residues also bring detrimental effect to the quality of graphene at the same time, as evidenced by the inhomogeneity of Raman signals (Figure S3, Supporting Information). On the other hand, for Cu–Ni alloy substrate, the polymeric residues were completely decomposed.^22^ As a result, both the nucleation density and defect of graphene were greatly reduced, leaving the *h*‐BN corners as the unique sites for graphene's heterogeneous nucleation.

Moreover, Cu–Ni substrates were also reported for a higher capacity of dissolving the introduced carbon atoms,^23^, ^30^, ^31^ which may also help facilitate the decrease of graphene nucleation density. Once the steady graphene nuclei were formed, carbon atoms were continuously supplied by both surface diffusion and bulk segregation and then boost the growth rate of graphene.^23^ The fast isothermal segregation growth of graphene therefore enables a shorter growth period, reduces the introduction of defects to the *h*‐BN grains, and eventually helps to make *h*‐BN and graphene both in high qualities.

Transmission electron microscopy (TEM) and selected area electron diffraction (SAED) measurements were used to study the lattice coherence between the graphene and the *h*‐BN grains. **Figure** **4**a,b shows the SEM and TEM images of the graphene/*h*‐BN heterostructure transferred onto TEM grid, respectively. Figure 4c–n presents SAED patterns taken from locations labeled in Figure 4b. It can be observed that each SAED pattern contains only one set of characteristic sixfold symmetric diffraction spots, indicating that all the measured *h*‐BN and graphene grains are well crystallized. Furthermore, although the three *h*‐BN grains (red labels) show different orientations with two parallel aligned and one rotated by 30°, graphene grains (blue labels) show exactly the same orientation as the *h*‐BN grains they stitch to. Such alignment was further confirmed by low energy electron microscopy (LEEM) and corresponding low energy electron diffraction (LEED) measurements (Figure S4, Supporting Information). The precise alignment of graphene to its matching *h*‐BN grains, its preferential nucleation uniquely at the corners of the *h*‐BN, together with literature studies^14^, ^16^ give strong indications of the epitaxial relationship between graphene and the attached *h*‐BN crystals.

**Figure 4:**
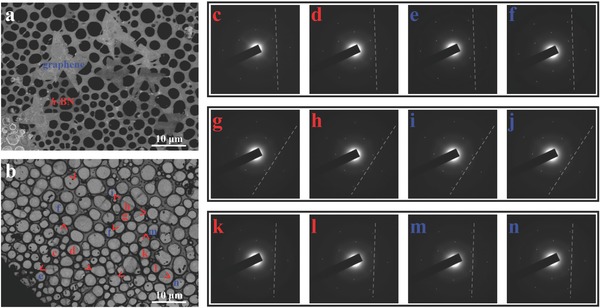
TEM and SAED characterizations showing the alignment of the lattice of graphene/*h*‐BN. a) SEM image of the graphene/*h*‐BN heterostructure grown on Cu–Ni after transferred onto TEM grid covered by ultrathin carbon film. b) TEM image of the same region in (a) with the corners of *h*‐BN grains marked. c–n) SAED patterns taken from the corresponding areas labeled in (b), either from the *h*‐BN grains (red labels) or the induced graphene grains (blue labels).

Auger electron spectroscopy (AES) was utilized to investigate the spatial distribution of specific elements in the graphene/*h*‐BN in‐plane heterostructure. **Figure** **5**a presents an SEM image of the heterostructure formed by growing *h*‐BN and graphene for 40 min and 75 s, respectively. Figure 5b shows the AES spectra taken from *h*‐BN (red dot) and graphene (blue dot), respectively, with the corresponding B (KLL), C (KLL), and N (KLL) spectra given in Figure 5c. It can be seen that the obvious B (KLL) and N (KLL) peaks locate at 176 and 385 eV on *h*‐BN grains respectively, together with a weak C (KLL) peak at 275 eV because of surface absorption. In addition, the graphene shows an intense carbon peak with no traces of boron or nitrogen detectable. Figure 5d–f shows the corresponding B (KLL), N (KLL), and C (KLL) maps of Figure 5a, respectively. All the three maps give homogenous element distribution inside graphene and the mating *h*‐BN grains together with sharp contrast at the interfaces, implying the existence of high‐quality in‐plane heterostructure. Similar characterizations were taken on graphene/*h*‐BN heterostructure synthesized on copper substrate (Figure S5, Supporting Information). It is found that both boron and nitrogen elements were detectable on exposed copper surface and even on graphene grains. More data about the characterizations on the graphene/*h*‐BN in‐plane heterostructure, including Raman maps, X‐ray photoemission spectroscopy (XPS), and optical absorption spectrum are presented in Figures S6 and S7 (Supporting Information), respectively.

**Figure 5:**
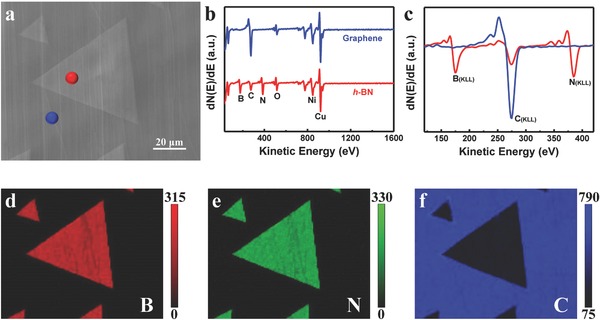
AES characterization confirming the elemental uniformity of the graphene/*h*‐BN in‐plane heterostructure. a) SEM image of the graphene/*h*‐BN in‐plane heterostructure obtained via 75 s of graphene growth following 40 min of *h*‐BN growth on Cu–Ni. b) Survey AES spectra taken in the dotted areas shown in (a). c) The spectra at the kinetic energy range from 120 to 420 eV. The corresponding d) B (KLL), e) N (KLL), and f) C (KLL) Auger electron maps obtained from the area shown in (a).

The success in synthesizing graphene and *h*‐BN both with high qualities brings in hope for various electrical and physical applications. For example, with an *h*‐BN grain serving as the dielectric substrate while the surrounding graphene serving as the bias electrodes, the transferred film provides an easy and effective platform to support phototransistors based on 2D semiconductor materials such as MoS_2_ (Figure S8, Supporting Information). Moreover, other types of interesting optoelectronic structures such as p–n junction devices could also utilize this platform. As shown in **Figure** **6**a, by using sequential transfer method,^32^ CVD‐grown monolayer WSe_2_ (p‐type) and MoS_2_ (n‐type) crystals were stacked on an *h*‐BN grain area while touching isolated parts of graphene. The structure is confirmed by Raman and photoluminescence (PL) spectra given in Figure 6b,c, respectively. The device exhibits a significant current recertification in *I*−*V* curve without illumination, while the corresponding current, especially the reverse current value, increases dramatically by increasing the illumination intensity, within a reasonably fast response time of ≈1 ms, as shown in Figure 6d–f. Both the high conductivity of graphene and the excellent dielectric property of *h*‐BN in the in‐plane heterostructure are thus well characterized and utilized, paving a way for further applications in logic and photoelectric circuits (Figure S9, Supporting Information).

**Figure 6:**
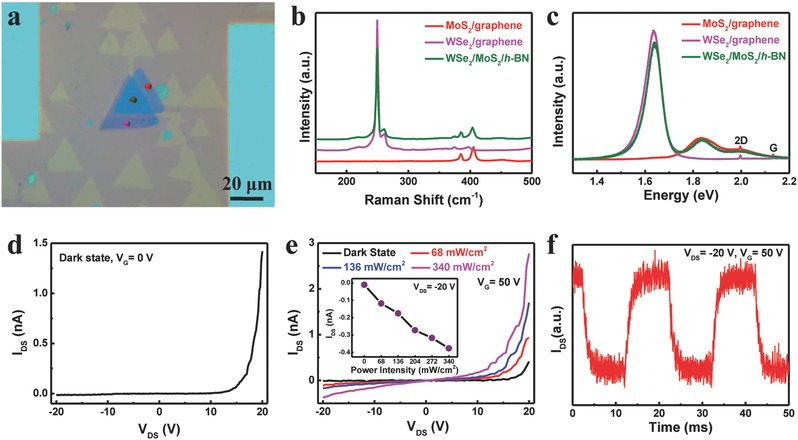
p–n junction photodetector fabricated on the transferred graphene/*h*‐BN in‐plane heterostructure. a) Optical image of the device. b) Raman spectra taken from the dotted areas in (a). c) PL spectra taken from the dotted areas in (a). d) *I*−*V* curve of the device in the dark. e) *I*−*V* characteristics of the device in the dark and under different illumination intensities with a gate bias set at 50 V. The inset shows the detected current as a function of illumination power intensity for *V*_DS_ = −20 V. f) Time‐resolved photoresponse (*V*_DS_: −20 V, illumination power intensity: 68 mW cm^−2^).

In summary, we have successfully synthesized graphene/*h*‐BN in‐plane heterostructure on Cu–Ni alloy by a two‐step LPCVD method. Graphene was found to preferentially nucleate at the corners of triangular *h*‐BN grains deposited in advance and follow the atomic orientation of *h*‐BN. Extensive characterizations confirm that the synthesized heterostructure is of high quality in crystallinity. Cu_85_Ni_15_, an alloy catalyst with higher catalytic power than copper, results in cleaner substrate surface, lower nucleation density, fast growth rate of graphene, and eventually large‐scale graphene/*h*‐BN in‐plane heterostructure. Based on the success, photodetector devices built on the unique platform were successfully demonstrated. This study may have significant impact on scalable synthesis of large area 2D heterostructures for future research and applications.

## Experimental Section

*Growth*: Cu–Ni alloy foils containing 15 atom% Ni (Cu_85_Ni_15_) were used as substrates, and time dependence of experimental parameters is given in Figure S1 (Supporting Information). Details of the substrate preparation and *h*‐BN growth were described in our earlier report.^22^ As *h*‐BN growth ended, the substrates were first maintained at 1050 °C for 10 min in an H_2_ flow at a low pressure to make sure that the borazane precursor was completely cooled down. Then an H_2_/CH_4_ (100/50 sccm) mixture were introduced for graphene growth. After that, the substrates cooled down to room temperature in a mixed Ar/H_2_ flow.

*Characterizations*: The graphene/*h*‐BN in‐plane heterostructures were characterized by SEM (Zeiss Supra55, operated at 3 kV), Raman spectroscopy (Renishaw, 514 or 532 nm laser wavelength), TEM (JEOL 2100F, 200 kV), and AES (PHI700, operated at 5 kV).

*MoS2 (WSe2) Transfer*: Graphene/*h*‐BN in‐plane heterostructure film grown on Cu–Ni was transferred onto SiO_2_/Si substrate first. CVD‐grown MoS_2_ crystals covered by a thin Poly (methyl methacrylate) (PMMA) film were lifted off in etching solution, washed in deionized water, and then picked up by a fresh SiO_2_/Si wafer. A fine tungsten probe was employed to cut a selected piece of PMMA/MoS_2_ out of the film and gently load it onto specific location shown in Figure 6a and S8a (Supporting Information). Finally, the whole sample was heated to 185 °C for 30 s and then the PMMA layer was washed off with acetone. WSe_2_ crystal was transferred using the same method.

*Device Fabrication and Measurement*: Device fabrication was done by e‐beam lithography, electron beam evaporation (Ti/Au), and lift‐off sequentially to contact graphene. Continuous graphene film was split by a tungsten probe. Electrical measurement was conducted in a home‐built probe station connected with Keithley 2634B source meter. Photocurrent was measured with a 543 nm laser (HeNe 543 nm, Melles Griot).

## Conflict of Interest

The authors declare no conflict of interest.

## Supporting information

Supplementary
