# Supplementary material for: Synthesis of High‐Quality Graphene and Hexagonal Boron Nitride Monolayer In‐Plane Heterostructure on Cu–Ni Alloy
Source: Adv Sci (Weinh). 2017 May 19;4(9):1700076. doi: 10.1002/advs.201700076 (PMC5604385; doi:10.1002/advs.201700076)
Supplement: Supplementary file 1 — Supplementary [file ADVS-4-na-s001.pdf]

Copyright WILEY-VCH Verlag GmbH & Co. KGaA, 69469 Weinheim, Germany, 2017.

Supporting Information

for *Adv. Sci.,* DOI: 10.1002/advs.201700076

Synthesis of High-Quality Graphene and Hexagonal Boron Nitride Monolayer In-Plane Heterostructure on Cu–Ni Alloy

Guangyuan Lu, Tianru Wu, Peng Yang, Yingchao Yang, Zehua Jin, Weibing Chen, Shuai Jia, Haomin Wang, Guanhua Zhang, Julong Sun, Pulickel M. Ajayan, Jun Lou, Xiaoming Xie,* and Mianheng Jiang*

Supporting Information

Synthesis of High-Quality Graphene and Hexagonal Boron Nitride Monolayer In-Plane Heterostructure on Cu-Ni Alloy

Guangyuan Lu, Tianru Wu, Peng Yang, Yingchao Yang, Zehua Jin, Weibing Chen, Shuai Jia, Haomin Wang, Guanhua Zhang, Julong Sun, Pulickel M. Ajayan, Jun Lou, * *Xiaoming Xie,* * *and Mianheng Jiang*

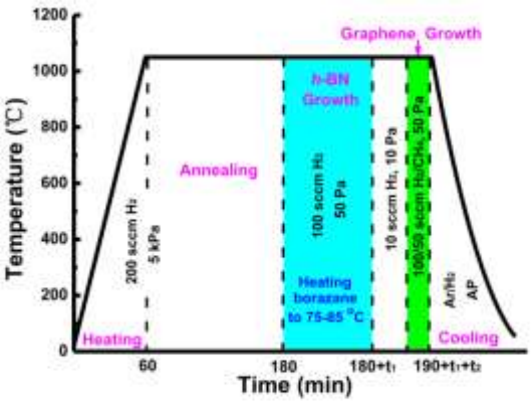

Figure S1. Process parameters for the growth of graphene/*h*-BN in-plane heterostructure on Cu-Ni substrate.

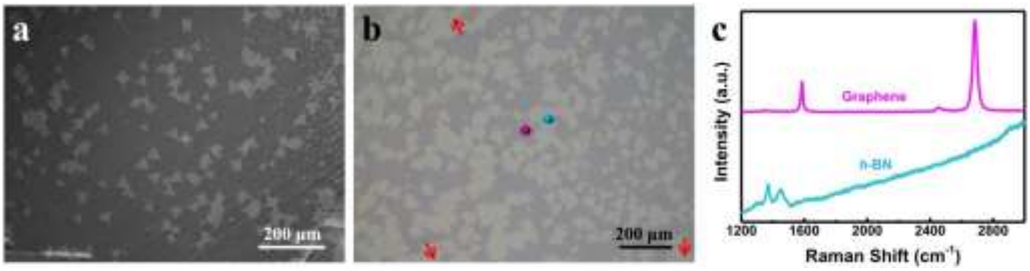

Figure S2. Large-area graphene/*h*-BN in-plane heterostructure. a) SEM image of the as-grown in-plane heterostructure on Cu-Ni. b) Optical image of a sample transferred onto a 90-nm SiO_2/Si substrate. In (b) graphene (magenta dot) shows darker color than *h*-BN grains (cyan dot) while the three red arrows point out the cracked areas of the film formed by transfer. c) Raman spectra taken from the marked areas with corresponding color dots in (b).

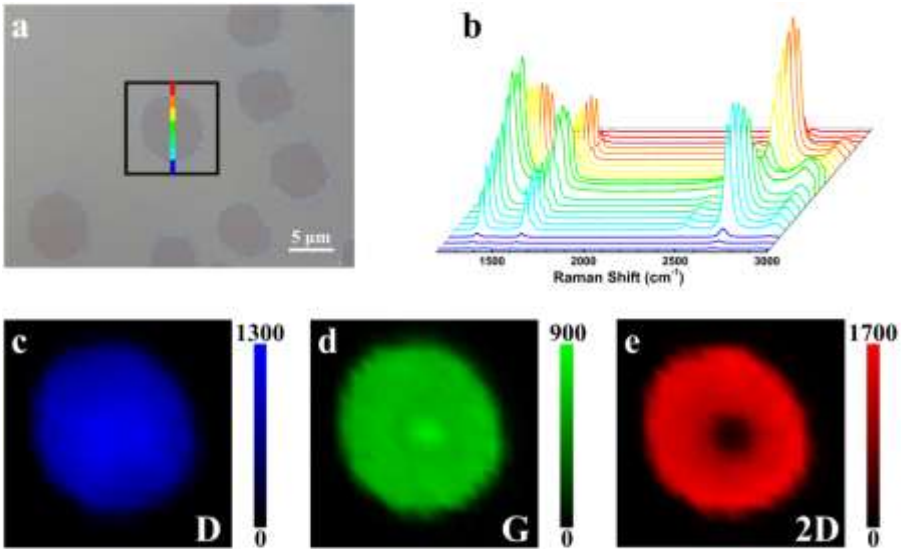

Figure S3. Raman characterization of graphene grain nucleated away from *h*-BN grains on copper surface. a) Optical image of the separate graphene grains on a 90-nm SiO_2/Si substrate. b) Raman spectra taken along the colorful line from red to blue shown in (a). c-e) Raman maps of D (c), G (d) and 2D (e) bands obtained from the square region marked in (a). It can be clearly seen that a much higher intensity ratio of D band to 2D band occurs at the center of the grain (spectra in green color in (b)).

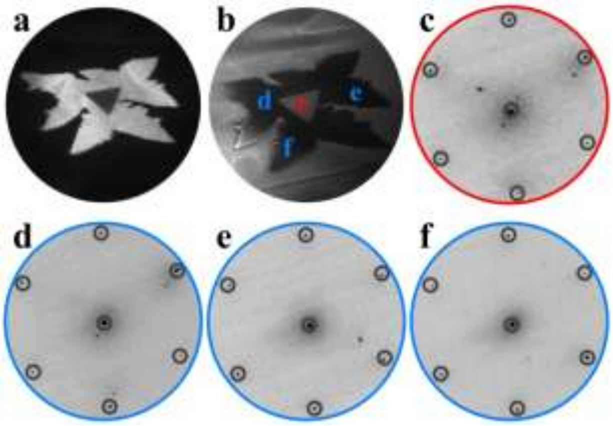

Figure S4. Lattice alignment investigated by LEEM and LEED. a) PEEM image of graphene/*h*-BN grains on Cu-Ni surface. b) LEEM image of the same grains. c-f) LEED patterns acquired at locations marked in (b). The diffraction spots of *h*-BN (c) and graphene (d,e,f) are circled in grey, all presenting the same orientation. The viewing fields in (a) and (b) are 60 and 50 μm, respectively.

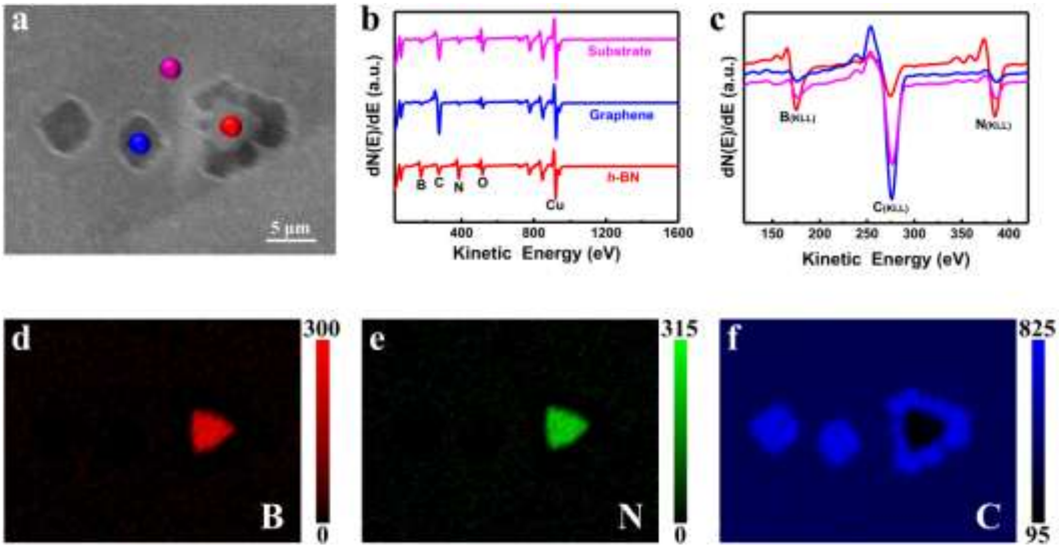

Figure S5. AES characterization of graphene/*h*-BN grains grown on copper surface. a) SEM image. b) Survey AES spectra taken in the dotted areas shown in (a). c) The spectra at the kinetic energy range from 120 to 420 eV. d-f) The corresponding B (KLL) (d), N (KLL) (e) and C (KLL) (f) Auger electron maps obtained from the area shown in (a). It can be seen that besides the *h*-BN grain, the exposed copper surface and the graphene grains also show distinguishable B (KLL) and N (KLL) peaks.

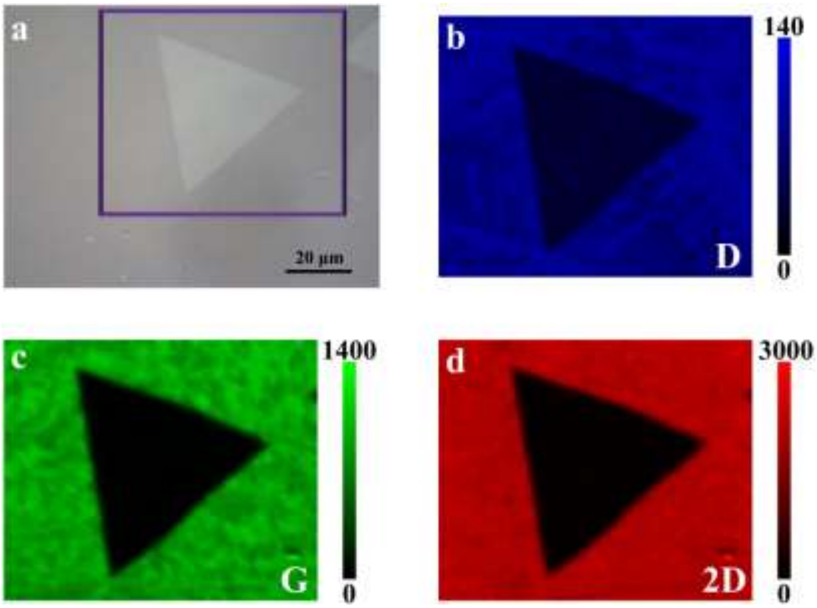

Figure S6. Raman maps of the graphene/*h*-BN in-plane heterostructure grown on Cu-Ni. a) Optical image of a sample transferred onto a 90-nm SiO_2/Si substrate. b-d) The corresponding Raman maps of D (b), G (c) and 2D (d) bands obtained from the region marked with purple rectangle in (a).

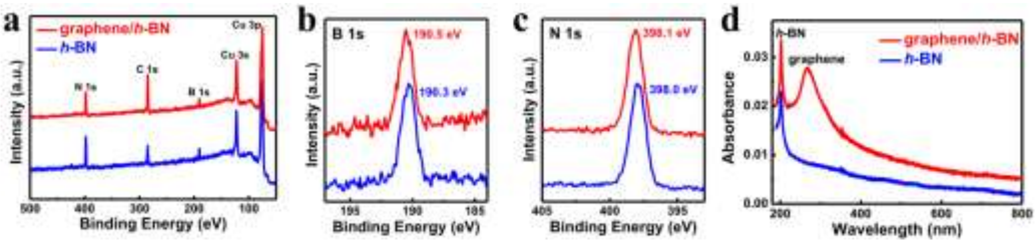

Figure S7. XPS and ultraviolet–visible absorption spectra. a) XPS spectra of *h*-BN grains and graphene/*h*-BN in-plane heterostructure on Cu-Ni alloy. b,c) The corresponding XPS spectra of B 1s (b) and N 1s (c) peaks demonstrating that the B-N bonds in *h*-BN were not destructed by the subsequent graphene growth. The B: N atomic ratios calculated through as-grown *h*-BN grains and graphene/*h*-BN in-plane heterostructure are 1.05:1 and 1.03:1, respectively. d) Ultraviolet-visible absorption spectra of *h*-BN grains and graphene/*h*-BN in-plane heterostructure transferred onto quartz substrates. The absorption peak at ~202 nm originates from the π-π* interband transition of *h*-BN, whereas the peak at ~269 nm describes the π-plasmon absorption of graphene.

.

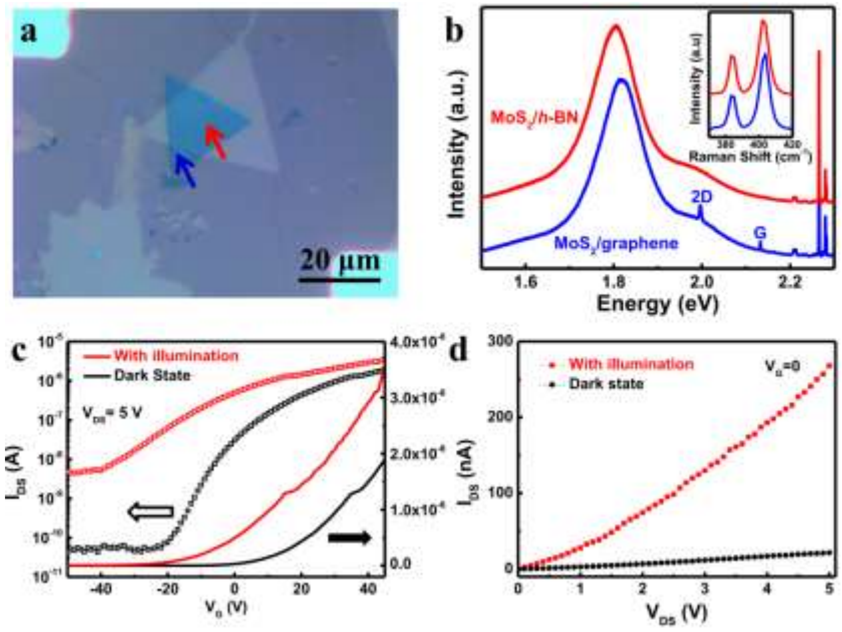

Figure S8. MoS_2 phototransistor based on the graphene/*h*-BN in-plane heterostructure. a) Optical image. b) PL spectra taken from the areas pointed out by the red and blue arrows in (a), respectively. The 2D and G Raman peaks from graphene are indicated, while the inset shows the corresponding E_{2g} and A_{1g} Raman peaks of MoS_2. c) Gate-tunable output characteristics of the device in dark and illuminated states. d) Drain-source (I_{DS}-V_{DS}) characteristics of the device in dark and illuminated states. It can be seen that the output current under the fixed bias voltage is dramatically increased by introducing illumination. The usage of the device as a photodetector is thus well proved.

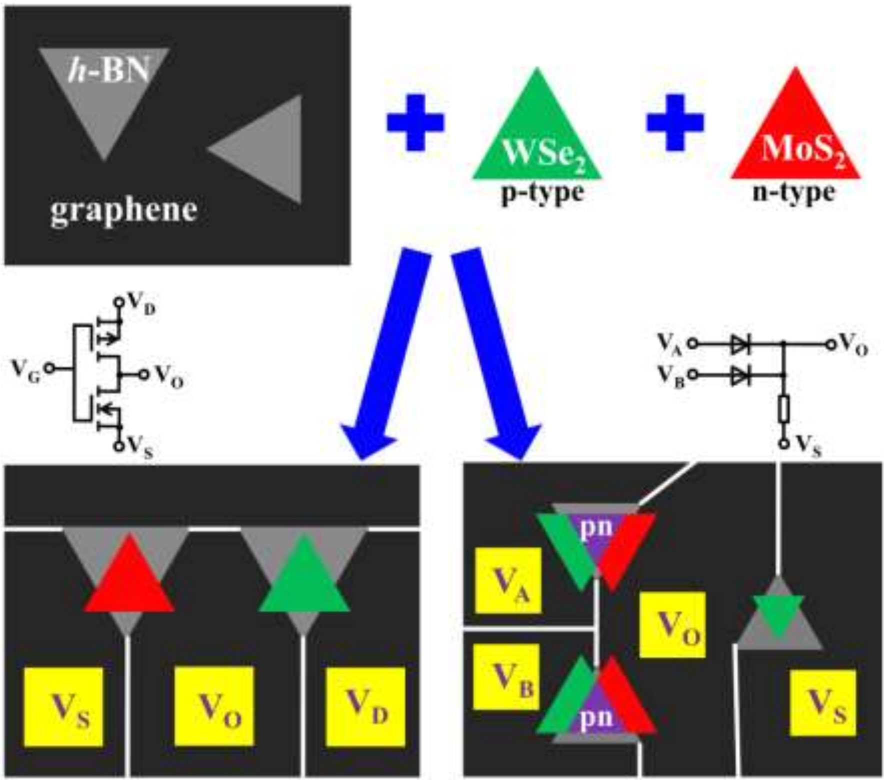

Figure S9. Schematic diagram of the logic circuits as CMOS (left) and "or" gate (right) based on our graphene/*h*-BN in-plane heterostructure and transition metal dichalcogenides.
